# A brain-inspired approach for SAR-to-optical image translation based on diffusion models

**DOI:** 10.3389/fnins.2024.1352841

**Published:** 2024-01-30

**Authors:** Hao Shi, Zihan Cui, Liang Chen, Jingfei He, Jingyi Yang

**Affiliations:** ^1^Radar Research Lab, School of Information and Electronics, Beijing Institute of Technology, Beijing, China; ^2^Beijing Institute of Technology Chongqing Innovation Center, Chongqing, China; ^3^National Key Laboratory of Science and Technology on Space-Born Intelligent Information Processing, Beijing, China

**Keywords:** synthetic aperture radar, SAR-to-optical image translation, brain-inspired approach, diffusion model, cognitive processes

## Abstract

Synthetic Aperture Radar (SAR) plays a crucial role in all-weather and all-day Earth observation owing to its distinctive imaging mechanism. However, interpreting SAR images is not as intuitive as optical images. Therefore, to make SAR images consistent with human cognitive habits and assist inexperienced people in interpreting SAR images, a generative model is needed to realize the translation from SAR images to optical ones. In this work, inspired by the processing of the human brain in painting, a novel conditional image-to-image translation framework is proposed for SAR to optical image translation based on the diffusion model. Firstly, considering the limited performance of existing CNN-based feature extraction modules, the model draws insights from the self-attention and long-skip connection mechanisms to enhance feature extraction capabilities, which are aligned more closely with the memory paradigm observed in the functioning of human brain neurons. Secondly, addressing the scarcity of SAR-optical image pairs, data augmentation that does not leak the augmented mode into the generated mode is designed to optimize data efficiency. The proposed SAR-to-optical image translation method is thoroughly evaluated using the SAR2Opt dataset. Experimental results demonstrate its capacity to synthesize high-fidelity optical images without introducing blurriness.

## 1 Introduction

Synthetic Aperture Radar (SAR) sensors are a crucial source of information for various applications such as target detection (Han et al., 2017, 2019, 2022; Deng et al., 2021; Tang et al., 2022), offering the ability to capture high-resolution data in all-time-all-weather conditions. However, interpreting SAR images requires specific expertise and practice. SAR images differ significantly from optical images as they do not align with the usual visual perceptions of the human eye. SAR images are complex, characterized by unique geometric features and speckle noise arising from SAR technology's intrinsic principles and imaging mechanisms. Therefore, as one of the feasible approach to assit interpreting SAR images, has been a growing trend toward translating SAR into optical images to aid in interpreting SAR images.

Generative Adversarial Networks (GANs) have become the leading models in translating SAR images into optical images. Originating from Goodfellow et al.'s (2014) concept, which drew inspiration from two-person zero-sum games in game theory, GANs have since evolved through various adaptations. Among these, Pix2Pix (Isola et al., 2017), a type of conditional GAN (Mirza and Osindero, 2014), has demonstrated considerable promise in image-to-image translation. Its most notable feature is the use of pairs of images for training. However, obtaining these paired images can be challenging, leading to the relevance of cycleGAN (Zhu et al., 2017), which operates with unpaired images. Researchers are now blending the strengths of both Pix2Pix and cycleGAN to enhance SAR-to-optical image translation, focusing on improvements in generators, discriminators, loss functions, and overall model structure. Improvements in generators often involve residual modules, dense connections, and dilated convolutions, aiming to enhance the interaction between encoders and decoders and preserve intricate details like color and texture (Ley et al., 2018; Turnes et al., 2020; Darbaghshahi et al., 2021; Fu et al., 2021; Guo et al., 2022; Shi et al., 2022; Yang et al., 2022). On the discriminator front, using multiple discriminators at different scales helps more accurately distinguish between translated and real images (Guo et al., 2022; Yang et al., 2022). In terms of loss functions, similarity metrics and perceptual loss are gaining traction (Hwang et al., 2020; Li et al., 2020; Darbaghshahi et al., 2021). As for the model structure, some studies also explore bidirectional generators, combining elements from Pix2Pix and cycleGAN, to enhance the quality of translated images (Wang et al., 2019; Fu et al., 2021; Shi et al., 2022). Despite these advancements, challenges persist due to the inherent differences between SAR and optical images. The translation network's feature extraction module often encounters information loss, and high-quality feature maps are hard to obtain. Besides, the scarcity of SAR-optical image pairs further hampers the training effectiveness of these models. While Pix2Pix-based models, relying on supervised learning, tend to yield higher-quality images, they demand more extensive datasets. In contrast, cycleGAN's unsupervised approach typically results in lower-quality images under similar conditions. Therefore, a balance between dataset availability and translation quality is crucial during model training. Moreover, while the zero-sum game concept of GANs is innovative, it poses significant challenges in training, especially for high-resolution images, as it tends to destabilize training and complicates adjustments and improvements.

Diffusion models have recently received considerable attention, which can be trained in a more stable way. The diffusion model was first proposed in 2015, and it was not until the introduction of the Denoising Diffusion Probabilistic Model (DDPM) (Ho et al., 2020) in 2020 that its development prospects gained widespread recognition. DDPM is a parameterized Markov chain that incrementally adds noise to data in a forward diffusion process until the original signal is wholly corrupted and then reconstructs the signal in a reverse diffusion process. While effective, DDPM's main limitation is the need for multiple iterations to produce high-quality images, unlike the single-pass requirement of GANs. To improve upon this, the Denoising Diffusion Implicit Model (DDIM) (Song et al., 2021) evolved from DDPM and introduced a non-Markov chain diffusion process. This innovation reduced the steps required in the inference process while maintaining sample quality. Further refinements came with the Improved DDPM (Nichol and Dhariwal, 2021), optimizing this process even more. The performance of diffusion models beat GAN for the first time in 2021 (Dhariwal and Nichol, 2021) when two researchers from OpenAI, Prafulla Dhariwal, and Alex Nichol, ingeniously refined the model architecture, resulting in a significant improvement in the quality of generated images. They also introduced classifier guidance for conditional image generation tasks, which was later streamlined by introducing classifier-free guidance (Ho and Salimans, 2021). This new approach eliminated the need for an external classifier by an equivalent structure, allowing direct conditional generation using a diffusion model. Regarding diffusion-based image translation, Palette (Saharia et al., 2022) has marked the competence of diffusion models. It follows a training approach akin to the GAN-based Pix2Pix, using paired image data. In contrast, UNIT-DDPM (Sasaki et al., 2021) utilized unpaired image data. Moreover, PITI (Wang et al., 2022) explored the use of pretraining to adapt diffusion models for various downstream image translation tasks. In summary, diffusion models have established themselves as capable alternatives to GANs for image generation and translation, offering more stable training and demonstrating a broadening scope of applications.

What's more, similar with pix2pix method, diffusion based method is also facing the challenge of lacking paired images in image traslation. The diffusion model is trained using a paired dataset of SAR and optical images. Note that the natural image processing often has access to billion-level image datasets, but as for remote sensing, the availability of SAR-optical image pairs is limited. Therefore, in the case of using SAR-optical image pairs, it is crucial to enhance the data utilization and avoid overfitting. Given that generative models serve as models for learning data distributions, once data augmentation is applied, the transformed images directly alter the target distribution represented by the original training samples, inevitably causing the generated sample distribution to deviate. To achieve reasonable data augmentation in generative models, the non-leaking data augmentation (Karras et al., 2020) has been proposed. It can prevent augmented style leaking into the target distribution represented by the original training samples. Furthermore, Patch Diffusion (Wang et al., 2023) introduced a generic patch-wise training framework to separate patches from full-size images and learn conditional loss functions on image patches. These methods improve the training performance under the condition of few samples.

In this paper, we employ a diffusion model in SAR to optical image translation and the main contributions can be summarized as follows:

1. A conditional diffusion model for SAR-to-optical image translation is proposed. This approach addresses the training instability encountered with GAN-based translation networks, particularly for high-resolution datasets. Our method leverages the diffusion model mechanism to avoid potential mode collapse issues during training, setting it apart from existing GAN-based image translation networks.
2. A feature map optimization method via self-attention and multi-level features fusion is proposed. This method enhances information extraction by compensating for the information loss, which is achieved through long skip connections derived from the U-Net architecture and a focus on global information via the self-attention mechanism derived from Vision Transformers. This approach allows for more effective source domain SAR image information extraction than existing image translation networks.
3. A data augmentation technique using non-leaking processing is proposed. This technique overcomes the limitation of lacking SAR-optical image pairs and does not compromise the integrity of the diffusion model. While augmenting SAR-optical image pairs, it incorporates matching the labels for these pairs to prevent damage to the translated images. In short, this technique distinguishes our approach from current image translation networks by applying data augmentation in generative models to avert overfitting in the translation network.

Experimental results demonstrate that diffusion models can effectively perform SAR-to-optical image translation, producing image quality on par with GAN-based models and excelling with high-resolution images.

## 2 Methods

**Figure 1:**
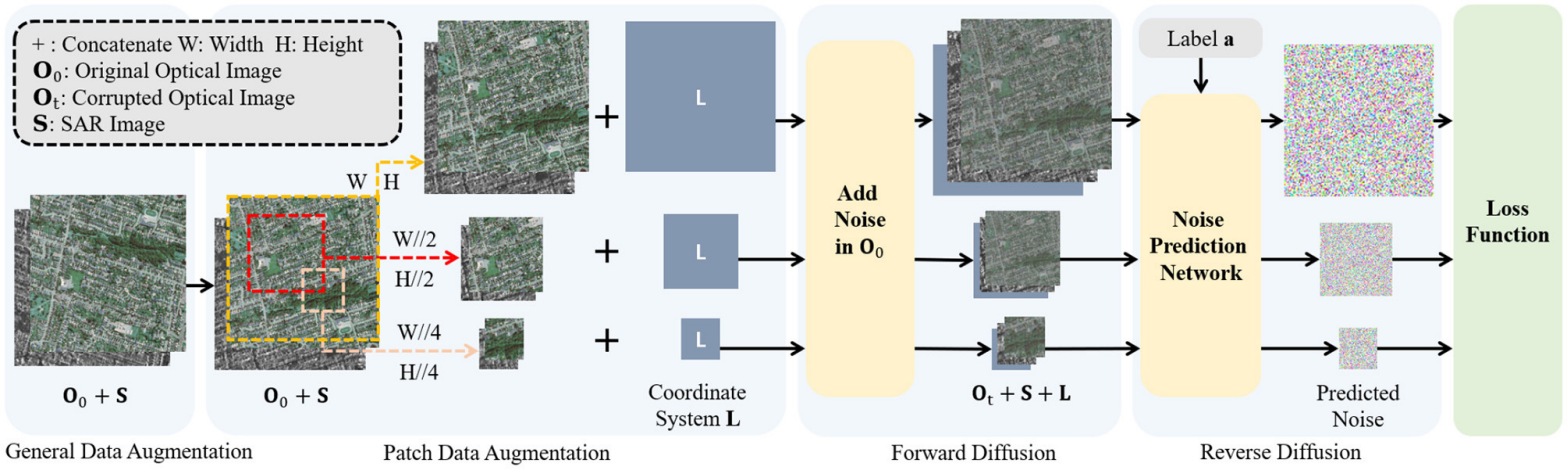
The overview of proposed framework from the perspective of data augmentation.

**Figure 2:**
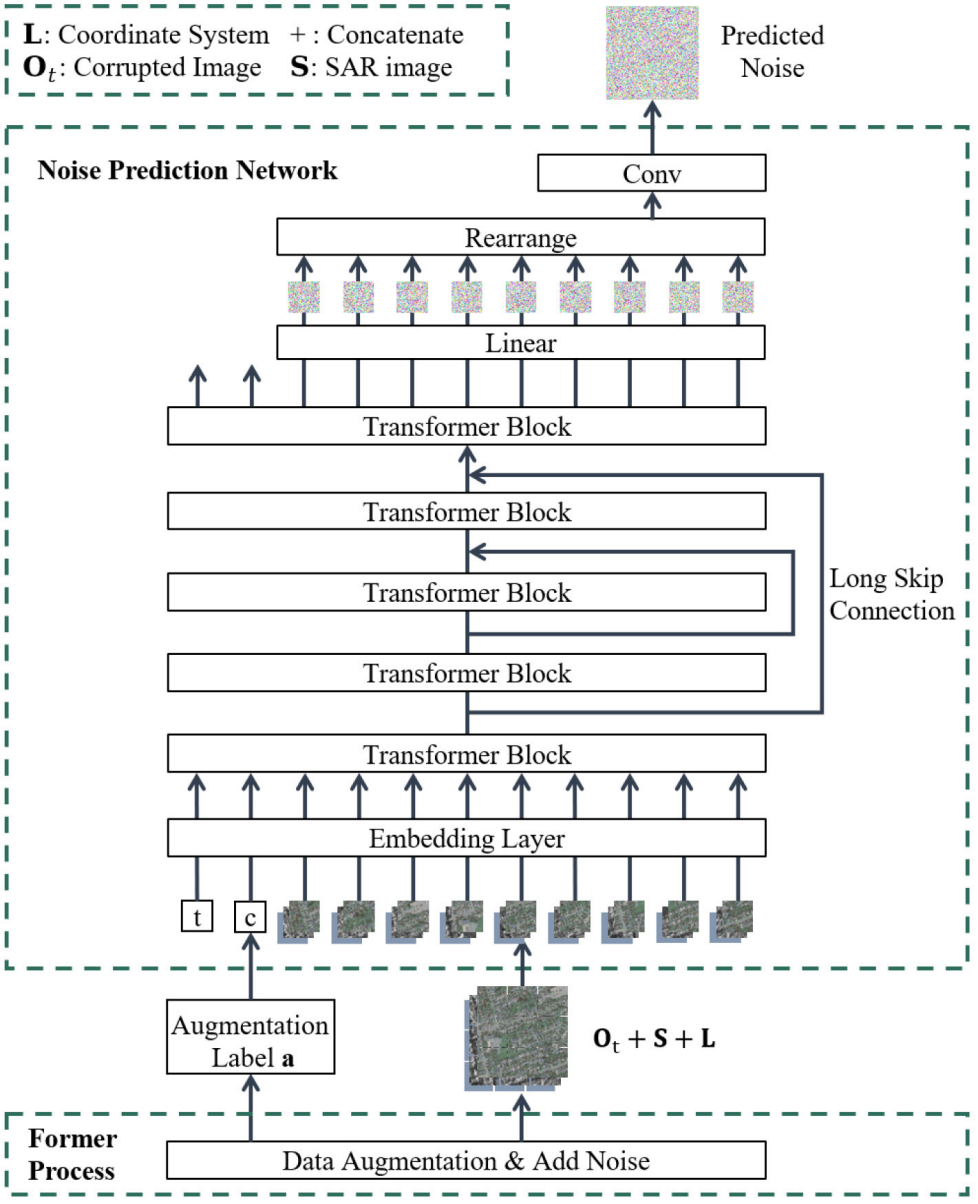
The overview of proposed framework from the perspective of noise prediction network structure.

The intuition behind the diffusion model comes from thermodynamics, where gas molecules diffuse from high-density to low-density areas, similar to the loss of information caused by noise interference. In the context of SAR-to-optical image translation, SAR and optical image respectively denoted as **S**, **O** ∈ ℝ^*H*×*W*×*C*^, where *H* and *W* refer to the height and width of the images, and *C* represents the number of channels in the images. To learn the data distribution of optical images, the model adds noise to optical images during the forward diffusion process and then samples from the distribution through the reverse diffusion process. Specifically, train the model to reconstruct optical images gradually during the reverse diffusion. Regarding the realization of conditional translation, the essence of SAR-to-optical image translation is an optical image generation task conditioned on SAR images. The input data consists of SAR-optical image pairs, which are concatenated along the channel dimension to provide reference information for SAR images. The SAR-optical image pairs are preprocessed and fed into the model, as shown in Figures 1, 2.

### 2.1 SAR-optical image diffusion

The SAR-optical image diffusion consists of two stages: forward diffusion and reverse diffusion, both of which are Markov chain processes.

In the forward diffusion process, Gaussian noise is gradually added to the given initial sample **O**_0_ ~ *q*(**O**) to generate noisy samples **O**_1_, **O**_2_, …, **O**_*T*_. The standard deviation of this noise is determined by the hyperparameter β_*t*_, and the mean is determined jointly by β_*t*_ and the data **O**_*t*_ at the current timestep *t*. β_*t*_ taken from the variable table ${\left\{\beta_{t}\in \left(0,1\right)\right\}}_{t=1}^{t}$. The mathematical expression of the forward diffusion process is shown in Equation (1).


(1) $$\begin{array}{l} \begin{array}{c} q(O_{t}|O_{t-1})=N(O_{t};\sqrt{1-\beta_{t}}O_{t-1},\beta_{t}I)\\ q(O_{1:T}|O_{0})=\prod_{t=1}^{T}q(O_{t}|O_{t-1}) \end{array} \end{array}$$

where, according to the reparameterization trick, *q*(**O**_*t*_) at any timestep can be computed based on **O**_0_ and β_*t*_ without iteration. Let α_*t*_ = 1 − β_*t*_, ${\bar{\alpha }}_{t}=\prod_{i=1}^{T}\alpha_{i}$ and the expression of *q*(**O**_*t*_|**O**_0_) can be derived as shown in Equation (2).


(2) $$q\left(O_{t}|O_{0}\right)=N\left(O_{t};\sqrt{{\bar{\alpha }}_{t}}O_{t-1},\left(1-{\bar{\alpha }}_{t}\right)I\right)$$

When *T* is sufficiently large, **O**_*T*_ ~ *q*(**O**_*T*_) can be considered to follow an independent Gaussian distribution, i.e., $q(O_{T})\sim N(O_{T};,I)$.

The sampling in the reverse diffusion process starts from the isotropic Gaussion noise **O**_*T*_, producing samples with progressively less noise **O**_*T*−1_, **O**_*T*−2_, …, until the final optical image sample **O**_0_ is obtained without noise. The process is based on the corresponding optical image **S**. Therefore, a parameter distribution *p*_θ_ is constructed to estimate *q*(**O**_*t*−1_|**O**_*t*_, **S**). The mathematical expression of the reverse diffusion process is shown in Equation (3).


(3) $$\begin{array}{l} p_{\theta }\left(O_{0:T}|S\right)=p\left(O_{T}\right)\prod_{t=1}^{T}p_{\theta }\left(O_{t-1}|O_{t},S\right)\\ p_{\theta }\left(O_{t-1}|O_{t},S\right)=N\left(O_{t-1};\mu_{\theta }\left(O_{t},S,t\right),\Sigma_{\theta }\left(O_{t},S,t\right)\right) \end{array}$$

For the variance **Σ**_θ_(**O**_*t*_, **S**, *t*), parameterize the variance as an interpolation between β_*t*_ and ${\tilde{\beta }}_{t}=\frac{1-\alpha_{t-1}}{1-\alpha_{t}}\beta_{t}$, as shown in Equation (4).


(4) $$\begin{array}{l} {\text{Σ}}_{\theta }\left({\text{O}}_{t},\text{S},t\right)=\exp\left(v\log\beta_{t}+\left(1-v\right)\log{\tilde{\beta }}_{t}\right) \end{array}$$

### 2.2 Architecture

The overall architecture of the model is shown in Figures 1, 2 in different perspectives. To begin with, corresponding data augmentation is achieved on the SAR-optical image pair. Then, in the reverse diffusion process, the noise added in the forward diffusion process is predicted by the constructed neural network ϵ_θ_, thereby translate the original SAR image **S** into corresponding optical image **O**_0_.

#### 2.2.1 Data augmentation

Before being fed into the translation network, the SAR-optical image pairs undergo two rounds of data augmentation.

Initially, six geometric augmentations, including flipping, scaling, and rotation, are applied with specified probabilities to the optical-SAR image pairs. Subsequently, in order to further increase the amount of data, a patch diffusion-based schedule shown in Equation (5) (Wang et al., 2023) is employed for random patch cropping on SAR-optical image pairs. The overall process of data augmentation is shown in Figure 1.


(5) $$(w,h)\sim p_{\left(w,h\right)}:=\{\begin{array}{ll} p, & when\;(w=W,h=H)\\ \frac{3}{5}(1-p), & when\;(w=W//2,h=H//2)\\ \frac{2}{5}(1-p), & when\;(w=W//4,h=H//4) \end{array}$$

In each training epoch, SAR-optical image pairs are cropped to different sizes depending on this schedule; put it in another way, each training image pair will be randomly cropped into three potential sizes. *p* is the ratio of training images that keep original size, *W, H* is the width and height of the original SAR-optical image pairs.

Then, noise is added to the optical images, and the SAR-optical image pairs are fed into the diffusion model for noise prediction. It is notable that after patch diffusion, original images are divideded into three different sizes, (*W, H*), (*W*//2, *H*//2), and (*W*//4, *H*//4) throughout whole training stage. Consequently, the calculation of loss will be employed in these three sizes. And then calculate the expectation together as the final loss.

For generative models, aggressive data augmentation can cause the model to learn the patterns introduced by the augmentation, deviating from the original distribution. To address this issue, in this work, when augmenting SAR and optical image pairs, the augmentation parameters are obtained synchronously as augmentation labels, indicating whether this pair patch with or without augmentation. For general data augmentation, the data augmentation label *a* is a 9-dimensional conditioning input vector, recording the applied data augmentation for each image pair, with 0 indicating no augmentation applied. *a* is inputted into the model in the form of category labels.

In addition, especially for patch diffusion augmentation, the location information *L* of the patch in the original image are used as augmentation labels. *L* is a pixel-level coordinate system with two channels, representing the pixel position coordinates (*x, y*) of the patch in the original image. By setting the upper left corner of the original image as (−1, −1) and the bottom-right corner as (1, 1), the pixel coordinate values are normalized relative to the original image size within the range of [−1, 1], resulting in a two-channel coordinate system. *L* and SAR-optical image pairs are inputted into the model via concatenation.

#### 2.2.2 Noise prediction network

Diffusion models often use U-Net as the noise prediction network ϵ_θ_. In the SAR-to-optical image translation task, the local geometric features of the SAR and optical images do not intuitively correspond. Fortunately, the self-attention module of ViT can globally attend to information, which is more conducive to learning the mapping relationship between SAR and optical images. U-ViT (Bao et al., 2023) is a simple and general backbone based on the ViT. Our model optimizes the extraction of feature maps through the U-ViT, parameterizing the noise prediction network ϵ_θ_, as shown in Figure 2. In terms of the advantages of applying the U-ViT, on one hand, following the core idea of ViT, U-ViT divides the input noise image **O**_*t*_, reference image **S** and location *L* into small blocks and flatten them as tokens, processing with timestep *t* and label *a* together. It uses self-attention instead of convolutions for information extraction. On the other hand, U-ViT adopts skip connections between shallow and deep layers, referencing the structure of U-Net, which can help the network better capture feature information at different levels.

### 2.3 Loss function

As mentioned before, the forward diffusion processing and the reverse diffusion processing could be formulated respectively as *q*(**O**_1:*T*_|**O**_0_) and *p*_θ_(**O**_0:*T*_|**S**). By organizing the negative log-likelihood function of the target data distribution, denoted as −log*p*_θ_(**O**_0:*T*_|**S**), a typical variational upper bound $L_{VLB}={𝔼}_{q\left({\text{O}}_{0:T}\right)}\left[\log\frac{q\left({\text{O}}_{1:T}|{\text{O}}_{0}\right)}{p_{\theta }\left({\text{O}}_{0:T}|\text{S}\right)}\right]$ can be obtained as shown in Equation (6).


(6) $$\begin{array}{l} L_{VLB}=\\ E_{q}[D_{KL}\left(q\left(x_{T}|x_{0}\right)||p_{\theta }\left(O_{T}\right)\right)+\\ \sum_{t=2}^{T}D_{KL}\left(q\left(O_{t-1}|O_{t},O_{0}\right)||p_{\theta }\left(O_{t-1}|O_{t},S\right)\right)-\log p_{\theta }\left(O_{0}|O_{1},S\right)] \end{array}$$

For two single-variable Gaussian distributions *q* and *p*, their KL divergence depends on the mean and variance of the distributions. Therefore, two branches of the neural network ϵ_θ_ are designed to fit the mean **μ**_θ_(**O**_*t*_, **S**, *t*) and variance **Σ**_θ_(**O**_*t*_, **S**, *t*) of the distribution *p*_θ_(**O**_*t*−1_|**O**_*t*_, **S**). As mentioned above, the conditions *a* and *L* related to data augmentation are also input into the network, so the neural network is denoted as ϵ_θ_(**O**_*t*_, **S**, *L, a, t*). To rearrange this term via integrating conditions as a function, ϵ_θ_ could be denoted further as ϵ_θ_(**O**_*t*_, *t*, **C**(**S**, *L, a*)).

To fit the mean **μ**_θ_(**O**_*t*_, *t*, **C**), involving unknown part the added noise, one branch of the network ϵ_θ_ outputs a random variable ϵ, representing the predicted noise. Using ϵ_θ_(**O**_*t*_, *t*, **C**) to estimate the noise component of a noisy sample **O**_*t*_, the loss function is shown in Equation (7).


(7) $$\begin{array}{l} L_{simple}\left(\theta \right):=E_{t,O_{0},S,\epsilon }\left[{\left||\epsilon -\epsilon_{\theta }\left(\sqrt{{\bar{\alpha }}_{t}}{\text{O}}_{0}+\sqrt{1-{\bar{\alpha }}_{t}}\epsilon ,t,\text{C}\right)|\right|}^{2}\right] \end{array}$$

This equation is equivalent to a noise prediction task. The noise prediction network is trained to minimize a noise prediction objective 𝔼[ϵ|**O**_*t*_, *t*, **C**].

To fit the variance **Σ**_θ_(**O**_*t*_, *t*, **C**), the other branch of the network ϵ_θ_ learns the coefficients *v* in the variance, obtaining *L*_*vb*_.

According to Equation (7), the loss function of the diffusion model calculates the similarity between the predicted noise and the added noise in the forward diffusion process. Besides, in this work, GAN and VGG losses are introduced to enhance the reality of the images, which could be denoted as *L*_*adv*_ and *L*_*perceptual*_, respectively. Finally, the integrated loss function is shown in Equation (8).


(8) $$\begin{array}{l} L=L_{simple}+\lambda_{1}L_{vb}+\lambda_{2}L_{perceptual}+\lambda_{3}L_{adv} \end{array}$$

where λ_1_, λ_2_, λ_3_ is hyperparameters, all set to 1 in this work.

### 2.4 Training and inference

During the forward diffusion process, the Gaussian noises are gradually added to **O**_0_ until obtaining the isotropic Gaussian noise **O**_*T*_. In the reverse diffusion process, a noise prediction network ϵ_θ_ is constructed to gradually obtain the pure optical translated **O**_0_ from **O**_*T*_ under the given conditions. At each denoising timestep, the corrupted optical image **O**_*t*_ and the coresponding condition **C**(**S**, *L, a*) are concatenated along the channel dimension. Then input them into the noise prediction network and get the predicted values of the added noise. During training, the model is optimized through iterative noise prediction. During sampling, the final translation result is progressively sampled according to the output of ϵ_θ_. The specific algorithms for training and sampling are shown in Algorithms 1, 2.

**Algorithm 1:** Training

1: **repeat**
2: **O**_0_~*q*(**O**)
3: *t*~*Uniform*({1, …, *T*})
4: ϵ~N(0,I)
5: Take gradient descent step on
*L* = *L*_*simple*_+λ_1_*L*_*vb*_+λ_2_*L*_*perceptual*_+λ_3_*L*_*adv*_,
where *L*_*simple*_(θ):=
$E_{t,O_{0},S,\epsilon }\left[{\left||\epsilon -\epsilon_{\theta }\left(\sqrt{{\bar{\alpha }}_{t}}{\text{O}}_{0}+\sqrt{1-{\bar{\alpha }}_{t}}\epsilon ,t,\text{C}\right)|\right|}^{2}\right]$
6: **until** converged

**Algorithm 2:** Sampling

1: $O_{T}\sim N(0,I)$
2: **for** *t* in {*T*, …, 1} **do**
3: z~N(0,I) if *t*>1, else **z** = **0**
4: ${\text{O}}_{t-1}=\frac{1}{\sqrt{\alpha_{t}}}\left({\text{O}}_{t}-\frac{1-\alpha_{t}}{\sqrt{1-{\bar{\alpha }}_{t}}}\epsilon_{\theta }\left({\text{O}}_{t},t,\text{C}\right)\right)+\sigma_{t}\text{z}$
5: **end for**
6: **return** **O**_0_

In addition, a hierarchical training strategy is adopted because high-resolution image translation significantly increases the model's parameters and pushes the computational cost to its limits. Specifically, a base model and an upsampler are trained separately. The base model learns the mapping relationship between SAR and optical images to translate small-sized images of 64 × 64. The upsampler then delineates the edges and details of the optical images using SAR images as guidance, resulting in high-resolution images of 512 × 512. The base model and upsampler employ distinct noise schedule and model structures to accommodate the generation and translation of images at different resolutions. The base model utilizes a cosine schedule, while the upsampler utilizes a linear schedule. The structure of the upsampler is lightweighted based on the base model, aiming to strike a balance between the accuracy of translated images and training efficiency.

## 3 Experiments

### 3.1 Dataset

We adopted the SAR2Opt dataset (Zhao et al., 2022) for our experiments. This dataset consists of SAR images collected by TerraSAR-X from 2007 to 2013 in ten cities across Asia, North America, Oceania, and Europe, with a spatial resolution of 1 m. Optical images were collected from Google Earth Engine and the paired SAR images through manual selection of control points. Image patches of size 600 × 600 pixels were extracted from the coregistered SAR-to-optical image pairs. The dataset contains a total of 2,077 paired large-sized, high-resolution SAR-optical image patches. We utilized the original paired dataset for training, randomly selecting 60% as the train set and allocating 20% each for the validation and test sets.

### 3.2 Evaluation metrics

To evaluate the effectiveness of image translation, we employed four metrics, namely, Peak Signal-to-Noise Ratio (PSNR), Structural Similarity (SSIM), complex wavelet Structural Similarity (CW-SSIM) and Frechet Inception Distance (FID). The first two metrics are popular methods for measuring the similarity between two images from different perspectives, the third is an improved method, while FID is the distance used to evaluate the performance of the model in image translation.

PSNR is one of the most commonly used and widely applied image evaluating metrics. This method assesses image quality based on the errors between corresponding pixels. SSIM evaluates image quality based on three different factors: brightness, contrast, and structure. The higher these two metrics, the more similar the translated image is to the real image.

CW-SSIM provides similarity evaluation of luminance change, contrast change and spatial translation insensitivity based on SSIM. Considering that it is unnecessary to blindly pursue pixel-by-pixel alignment of the image in the style translation task, subtle style changes do not affect the human eye's interpretation of the image. Therefore, CW-SSIM is introduced as one of the evaluation indexes in this work. Similar to SSIM, higher CW-SSIM values indicate greater image similarity.

FID is a measure used to measure the distance between the translated image distribution and the real image distribution. Firstly, the Inception network is used to extract features, and then a Gaussian model is used to model the feature space, and finally, the distance between two features is calculated. A lower FID means higher image quality and diversity.


(9) $$\begin{array}{l} PSNR=10\log_{10}\frac{MAX_{I}^{2}}{MSE} \end{array}$$

(10) $$\begin{array}{l} SSIM\left(x,y\right)=\frac{\left(2\mu_{x}\mu_{y}+c_{1}\right)\left(2\sigma_{xy}+c_{2}\right)}{\left(\mu_{x}^{2}+\mu_{y}^{2}+c_{1}\right)\left(\sigma_{x}^{2}+\sigma_{y}^{2}+c_{2}\right)} \end{array}$$

(11) $$\begin{array}{l} \tilde{S}\left({\text{c}}_{x},{\text{c}}_{y}\right)=\frac{2|\sum_{i=1}^{N}c_{x,i}c_{y,i}^{*}|+K}{\sum_{i=1}^{N}{\left|c_{x,i}\right|}^{2}+\sum_{i=1}^{N}{\left|c_{y,i}\right|}^{2}+K} \end{array}$$

(12) $$\begin{array}{l} FID\left(x,y\right)=||\mu_{x}-\mu_{y}|{|}_{2}^{2}+Tr\left(C_{x}+C_{y}-2{\left(C_{x}C_{y}\right)}^{\frac{1}{2}}\right) \end{array}$$

The calculation formulas for PSNR, SSIM, CW-SSIM, and FID are respectively shown in Equations (9, 10, 11, and 12). In Equation (9), *MAX*_*I*_ denotes the maximum possible pixel value of the image. *MSE* denotes mean squared error between images *x* and *y*. In Equation (10), μ_*x*_, μ_*y*_, σ_*x*_, σ_*y*_, σ_*xy*_ denote the means, variances, and covariance of the images *x* and *y*. *c*_1_, *c*_2_ are constants. In Equation (11), **c**_*x*_ = {*c*_*x, i*_|*i* = 1, …, *N*} and **c**_*y*_ = {*c*_*y, i*_|*i* = 1, …, *N*} denote two sets of coefficients extracted at the same spatial location in the same wavelet subbands of the two images being compared. c^*^ is the complex conjugate of *c*. *K* is a small positive constant. In Equation (12), μ_*x*_, μ_*y*_, *C*_*x*_, *C*_*y*_ denote the means and covariance matrices of the image features. *Tr*(·) is the trace of matrix.

### 3.3 Result

**Figure 3:**
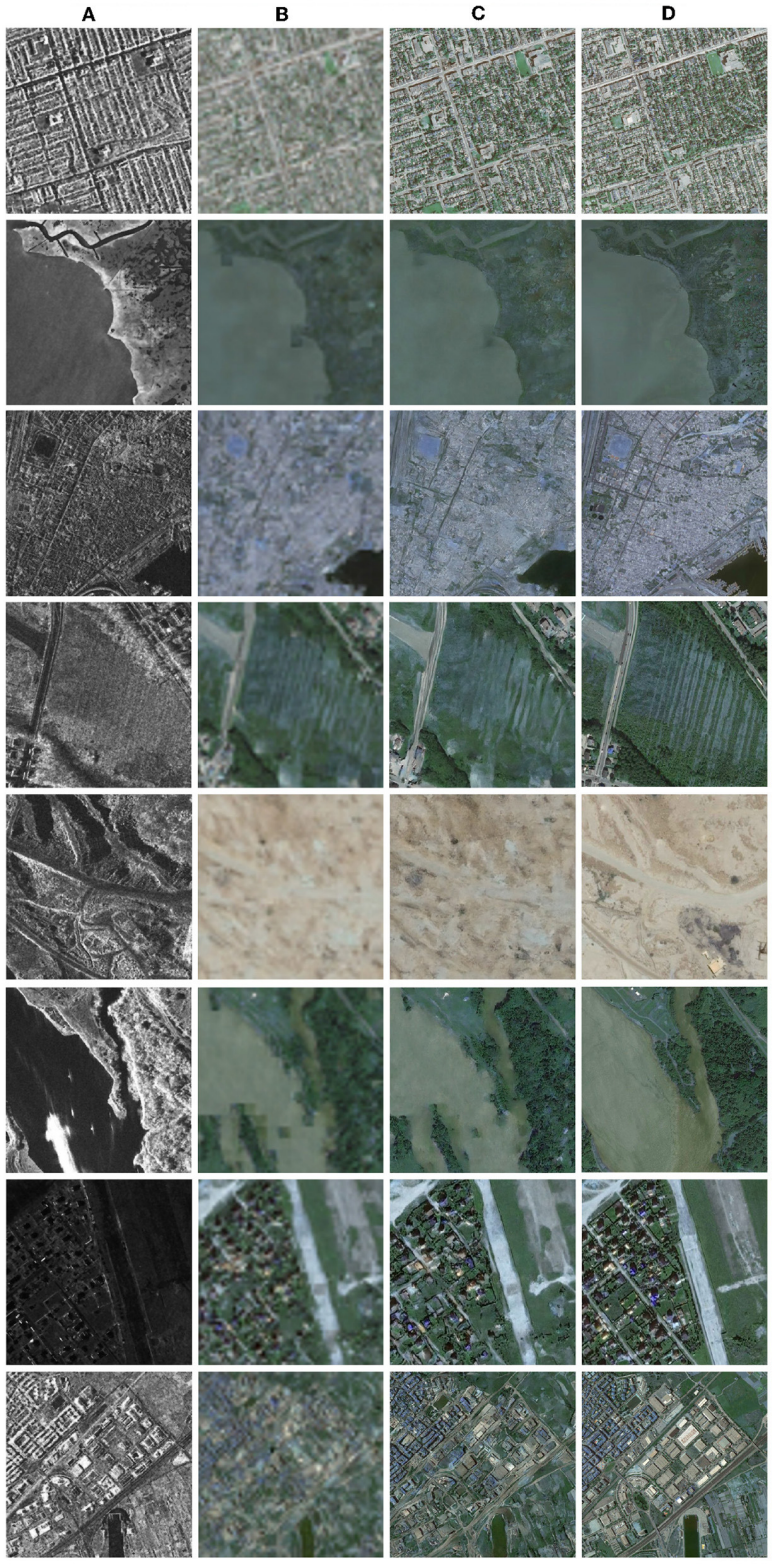
Experimental results in different images. **(A)** Reference SAR images. **(B)** Low-resolution (64 × 64) translation results in the first stage. **(C)** High-resolution(512 × 512) translation results in the second stage. **(D)** Ground truth of optical images.

The Figure 3 presents some exemplary results of the network based on the SAR2Opt dataset, with each column from left to right representing SAR images, translated low resolution images, translated high-resolution images, and real optical images. The results demonstrate that the proposed diffusion model is capable of performing SAR to optical image translation tasks. The translated images exhibit sharp edges, clear details, and successfully reproduce the texture of optical images. The reality achieved makes it difficult to distinguish the translated images from real images with human eyes. For regions with abundant data in the dataset, such as urban and suburban areas, the diffusion model accurately reproduces the terrain classification for each pixel. Even for areas with limited data, such as ports and airports, the diffusion model can still generate identifiable terrain features. Overall, the proposed model effectively accomplished SAR to optical image translation, thereby providing valuable assistance in the interpretation of SAR images.

However, there are some mistranslations of small elements such as ships and aircraft, which may be due to the lack of relevant examples in the training set. In addition, it was notable that the moving targets could lead to the difference between paired SAR and optical images. As shown in row sixth of the Figure 3, the ship appearing in the SAR image was not displayed in the optical image, which also led to the model's misjudgment of moving targets. For such targets, specialized datasets are still needed to achieve better translation performance.

### 3.4 Ablation experiment

**Figure 4:**
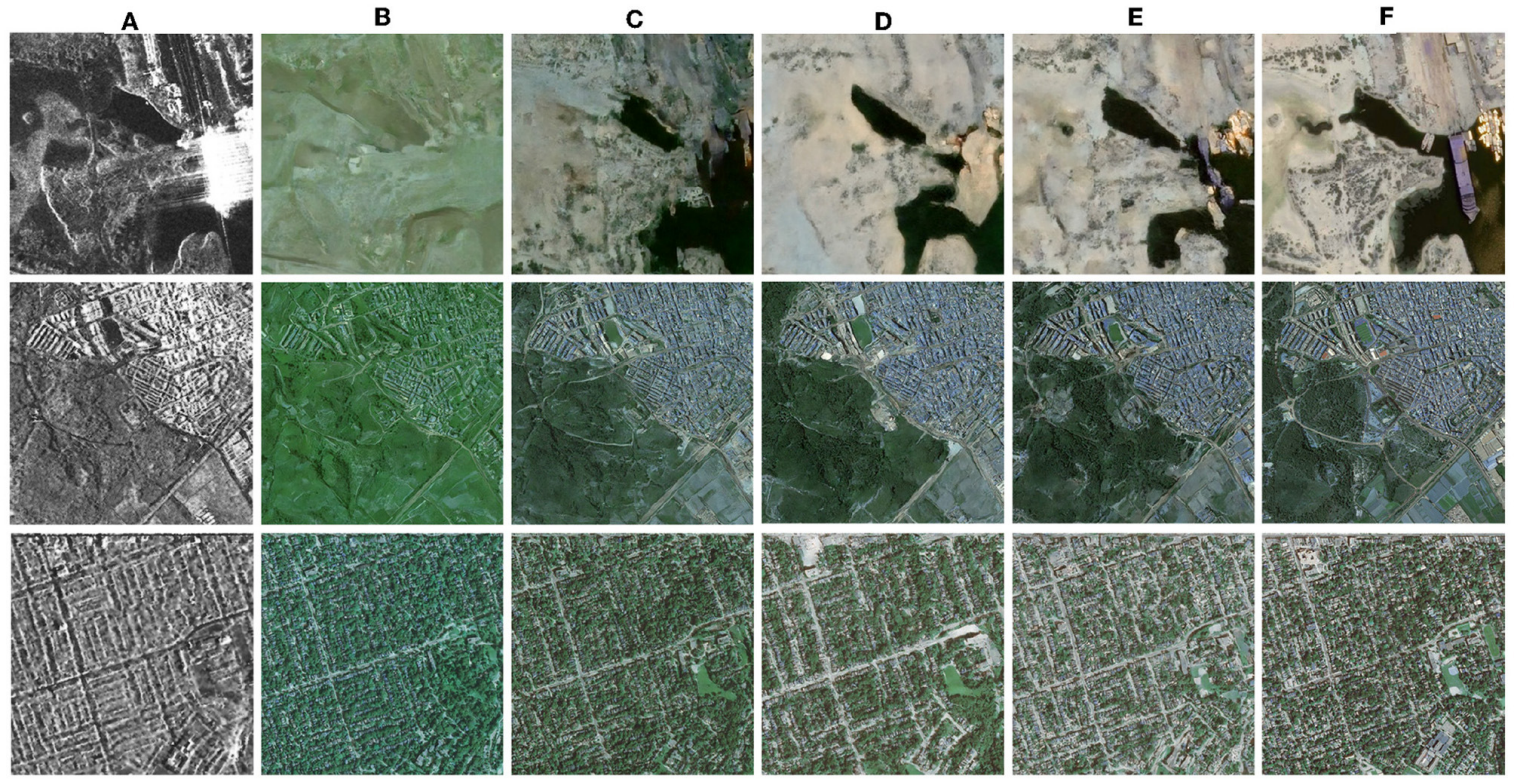
Ablation results. **(A)** Reference SAR images. **(B)** Palette. Baseline. **(C)** U-ViT diffusion model. Apply the U-ViT structure only. **(D)** Data argument model. Apply data augmentation only. **(E)** Proposed model. Apply the U-ViT structure and data augmentation. **(F)** Ground truth (optical images).

To validate the effectiveness of the model structure U-ViT and the data augmentation, the qualitative results of the ablation experiment are shown in Figure 4, and the quantitative results are shown in Table 1.

**Table 1:** Ablation experiment results in main metrics.

**Structure**	**PSNR**	**SSIM**	**CW-SSIM**	**FID**
Palette	14.80	0.2719	0.4474	182.38
U-ViT diffusion model	15.47	0.2846	0.4898	171.76
Data augmentation model	15.40	**0.2914**	0.4981	179.11
Proposed	**15.93**	0.2831	**0.5086**	**170.26**

The bold values represent the best result for a given metric.

Palette is a general diffusion-based image-to-image translation model, therefore it is used as the baseline. However, it was found in the experiment that color shift occurred under limited samples and small batch size conditions. Our proposed method can eliminate color shift under the same conditions. It is more suitable for specialized tasks of SAR to optical image translation.

From the qualitative results, it can be seen that the excellent feature extraction ability of U-ViT enables the noise prediction network to recover finer edges and details. As shown in the third column in Figure 4, when using the U-ViT noise prediction network, inconspicuous narrow roads become clear and visible. The data augmentation significantly improves the quality of image translation, as shown in the fourth column in Figure 4, especially for terrain categories with fewer samples, such as the yellow category. In the results without data augmentation, the style of the yellow category is almost absent, while reasonable colors are generated after applying data augmentation. When the two technologies are combined to obtain the proposed model, as shown in the fifth column of Figure 4, the content and texture of the image are well translated.

From the quantitative results, it can be seen that the proposed model structure U-ViT and data augmentation can both improve the similarity between translated images and real images. When two methods are combined, better results can be obtained. It should be noted that SSIM is based on MSE regression techniques, which tend to be conservative for high-frequency details. Sometimes images with blurred details can actually achieve higher SSIM, just like the results after only data augmentation. Therefore, we believe that the evaluation of CW-SSIM has better reference value. CW-SSIM is not sensitive to small offset, which is more in line with the human eye's habit of interpreting high-resolution images.

Overall, the data augmentation and U-ViT both enhance the quality of the generated images and alleviate the demand for large-scale datasets in diffusion models.

### 3.5 Comparison experiment

**Figure 5:**
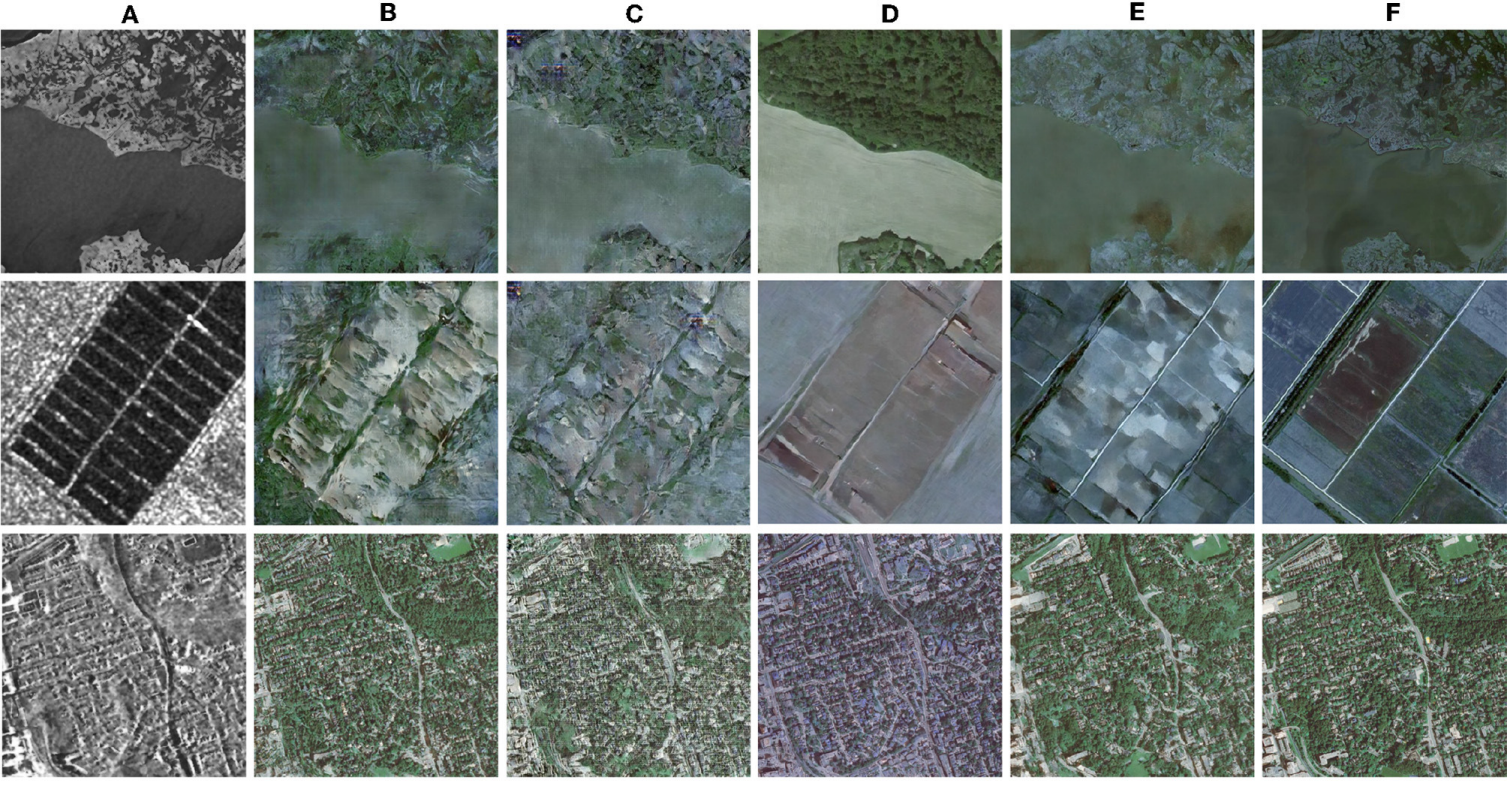
Comparison experiment results of four methods. **(A)** Reference SAR images. **(B)** Pix2Pix. **(C)** Pix2PixHD. **(D)** Palette. **(E)** Proposed model. **(F)** Ground truth (optical images).

The proposed model is compared with typical conditional generative networks and SAR-to-optical translation networks, and the results are shown in Figure 5 and Table 2. We set the recommended parameters for each network as stated in their respective papers and selected the best test results after multiple trainings. We compare all experiments using the same set of images.

**Table 2:** Comparison experiment results in main metrics.

**Structure**	**PSNR**	**SSIM**	**CW-SSIM**	**FID**
Pix2Pix	14.95	0.2282	0.4854	216.89
Pix2PixHD	14.70	0.2041	0.4760	219.06
Palette	14.80	0.2719	0.4274	182.38
Proposed	**15.93**	**0.2831**	**0.5086**	**170.26**

The bold values represent the best result for a given metric.

From the Figure 5, it can be observed that most networks designed for low-resolution images perform poorly on high-resolution images and face challenges in achieving convergence. Even for pix2pixHD, which is specifically designed for high-resolution image generation, there are still instances of blurriness and artifacts in the results. In fact, networks based on conditional GANs often fail to produce convincing high-resolution images, possibly due to the introduced *L*_1_ loss, which only tends to encourage low-frequency features. In contrast, our proposed model not only capture low-frequency features but also effectively acquire high-frequency features, resulting in images that closely resemble optical images. Furthermore, our proposed model outperforms the Palette, indicating its suitability for SAR-to-optical image translation tasks.

The quantitative results are shown in the table. Compared with the GAN-based model, the diffusion model-based results obtained better FID, indicating a closer distance between the distribution of translated images and the real images. Compared with Palette, the improved method proposed in this paper successfully makes the basic diffusion model more suitable for SAR to optical image translation tasks, improving PSNR and SSIM.

## 4 Conclusion

In this paper, the diffusion model is employed to accomplish the SAR-Optical images translation, assisting in the interpretation of SAR image. In experiments on the high-resolution data set, the stability of SAR to optical image translation model training is guaranteed, and the image translation quality was better than that of GAN-based methods. The proposed model combines long-skip connections and self-attention mechanisms to optimize feature maps, enhance the feature extraction capabilities of the model. This architectural style results in a better translation of the edges, details and overall texture of the image. Data augmentation implemented on SAR-optical image pairs increases the training sample size of SAR-optical images while avoiding leakage of augmentation patterns into translated images. This significantly improves the quality of translation for patterns with smaller sample sizes. Experimental results evaluated on the SAR2Opt dataset demonstrate that our approach achieves state-of-the-art performance.

On the basis of the current experiments, there are still some further experiments that could be carried out. First, the diffusion model requires multiple iterations to complete the translation of the images, unlike GAN which only requires one. Therefore, how to improve the translation efficiency of the diffusion model-based method is an important topic. Second, the proposed method has only been tested on the SAR2Opt data set, and its general applicability to different types of data sets remains to be explored. Third, the current research on SAR to optical image translation is still limited to the style translation of terrain, which is determined by the existing data sets. Reasonable other target data sets should continue to be constructed to promote the SAR to optical translation technology to more fields.

## Data availability statement

Publicly available datasets were analyzed in this study. This data can be found here: https://github.com/MarsZhaoYT/Sar2Opt-Heterogeneous-Dataset.

## Author contributions

HS: Conceptualization, Writing – original draft, Writing – review & editing. ZC: Conceptualization, Formal analysis, Methodology, Writing – original draft. LC: Conceptualization, Writing – review & editing. JH: Methodology, Writing – review & editing. JY: Formal analysis, Methodology, Writing – review & editing.

## References

[B1] Bao F. Nie S. Xue K. Cao Y. Li C. Su H.. (2023). “All are worth words: a vit backbone for diffusion models, in *2023 IEEE/CVF Conference on Computer Vision and Pattern Recognition (CVPR)* (Vancouver, BC), 22669–22679. 10.1109/CVPR52729.2023.02171

[B2] Darbaghshahi F. N. Mohammadi M. R. Soryani M. (2021). Cloud removal in remote sensing images using generative adversarial networks and sar-to-optical image translation. IEEE Trans. Geosci. Remote Sens. 60, 1–9. 10.1109/TGRS.2021.3131035

[B3] Deng C. He S. Han Y. Zhao B. (2021). Learning dynamic spatial-temporal regularization for UAV object tracking. IEEE Signal Process. Lett. 28, 1230–1234.

[B4] Dhariwal P. Nichol A. (2021). Diffusion models beat gans on image synthesis. Adv. Neural Inf. Process. Syst. 34, 8780–8794. 10.48550/arXiv.2105.05233

[B5] Fu S. Xu F. Jin Y.-Q. (2021). Reciprocal translation between sar and optical remote sensing images with cascaded-residual adversarial networks. Sci. China Inf. Sci. 64, 1–15. 10.1007/s11432-020-3077-5

[B6] Goodfellow I. J. Pouget-Abadie J. Mirza M. Xu B. Warde-Farley D. Ozair S.. (2014). “Generative adversarial nets,” in Proceedings of the 27th International Conference on Neural Information Processing Systems - Volume 2 (Cambridge, MA: MIT Press), 2672–2680.

[B7] Guo Z. Guo H. Liu X. Zhou W. Wang Y. Fan Y.. (2022). Sar2color: learning imaging characteristics of sar images for sar-to-optical transformation. Remote Sens. 14:3740. 10.3390/rs14153740

[B8] Han Y. Deng C. Zhang Z. Li J. Zhao B. (2017). “Adaptive feature representation for visual tracking,” in 2017 IEEE International Conference on Image Processing (ICIP) (IEEE), 1867–1870.

[B9] Han Y. Deng C. Zhao B. Tao D. (2019). State-aware anti-drift object tracking. IEEE Trans. Image Process. 28, 4075–4086. 30892207 10.1109/TIP.2019.2905984

[B10] Han Y. Liu H. Wang Y. Liu C. (2022). A comprehensive review for typical applications based upon unmanned aerial vehicle platform. IEEE J. Select. Topics Appl. Earth Observ. Remote Sens. 15, 9654–9666.

[B11] Ho J. Salimans T. (2021). “Classifier-free diffusion guidance,” in *NeurIPS 2021 Workshop on Deep Generative Models and Downstream Applications* (Montreal, QC).

[B12] Ho J. Jain A. Abbeel P. (2020). Denoising diffusion probabilistic models. Adv. Neural Inf. Process. Syst. 33, 6840–6851. 10.48550/arXiv.2006.11239

[B13] Hwang J. Yu C. Shin Y. (2020). “Sar-to-optical image translation using ssim and perceptual loss based cycle-consistent gan,” in *2020 International Conference on Information and Communication Technology Convergence (ICTC)* (Jeju: IEEE), 191–194. 10.1109/ICTC49870.2020.9289381

[B14] Isola P. Zhu J.-Y. Zhou T. Efros A. A. (2017). “Image-to-image translation with conditional adversarial networks,” in *Proceedings of the IEEE Conference on Computer Vision and Pattern Recognition* (Honolulu, HI: IEEE), 1125–1134. 10.1109/CVPR.2017.632

[B15] Karras T. Aittala M. Hellsten J. Laine S. Lehtinen J. Aila T.. (2020). Training generative adversarial networks with limited data. Adv. Neural Inf. Process. Syst. 33, 12104–12114. 10.48550/arXiv.2006.06676

[B16] Ley A. Dhondt O. Valade S. Haensch R. Hellwich O. (2018). “Exploiting gan-based sar to optical image transcoding for improved classification via deep learning,” in *EUSAR 2018*; 12th European *Conference on Synthetic Aperture Radar* (VDE) (Aachen), 1–6.

[B17] Li Y. Fu R. Meng X. Jin W. Shao F. (2020). A sar-to-optical image translation method based on conditional generation adversarial network (cgan). IEEE Access 8, 60338–60343. 10.1109/ACCESS.2020.2977103

[B18] Mirza M. Osindero S. (2014). Conditional generative adversarial nets. Comput. Sci. 2672–2680. 10.48550/arXiv.1411.1784

[B19] Nichol A. Q. Dhariwal P. (2021). “Improved denoising diffusion probabilistic models,” in *International Conference on Machine Learning* (PMLR) (New York, NY), 8162–8171.

[B20] Saharia C. Chan W. Chang H. Lee C. Ho J. Salimans T.. (2022). “Palette: image-to-image diffusion models,” in *ACM SIGGRAPH 2022 Conference Proceedings* (New York, NY: ACM), 1–10. 10.1145/3528233.3530757

[B21] Sasaki H. Willcocks C. G. Breckon T. P. (2021). UNIT-DDPM: unpaired image translation with denoising diffusion probabilistic models. arXiv. [Preprint]. 10.48550/arxiv.2104.05358

[B22] Shi H. Zhang B. Wang Y. Cui Z. Chen L. (2022). Sar-to-optical image translating through generate-validate adversarial networks. IEEE Geosci. Remote Sens. Lett. 19, 1–5. 10.1109/LGRS.2022.3168391

[B23] Song J. Meng C. Ermon S. (2021). “Denoising diffusion implicit models,” in *International Conference on Learning Representations* (Ithaca, NY).

[B24] Tang L. Tang W. Qu X. Han Y. Wang W. Zhao B. (2022). A scale-aware pyramid network for multi-scale object detection in SAR images. Remote Sens. 14:973.

[B25] Turnes J. N. Castro J. D. B. Torres D. L. Vega P. J. S. Feitosa R. Q. Happ P. N.. (2020). Atrous cGAN for SAR to optical image translation. IEEE Geosci. Remote Sens. Lett. 19, 1–5. 10.1109/LGRS.2020.3031199

[B26] Wang L. Xu X. Yu Y. Yang R. Gui R. Xu Z.. (2019). Sar-to-optical image translation using supervised cycle-consistent adversarial networks. IEEE Access 7, 129136–129149. 10.1109/ACCESS.2019.2939649

[B27] Wang T. Zhang T. Zhang B. Ouyang H. Chen D. Chen Q.. (2022). Pretraining is all you need for image-to-image translation. arXiv. [Preprint]. 10.48550/arxiv.2205.12952

[B28] Wang Z. Jiang Y. Zheng H. Wang P. He P. Wang Z.. (2023). Patch diffusion: faster and more data-efficient training of diffusion models. arXiv. [Preprint]. 10.48550/arxiv.2304.12526

[B29] Yang X. Wang Z. Zhao J. Yang D. (2022). FG-GAN: a fine-grained generative adversarial network for unsupervised sar-to-optical image translation. IEEE Trans. Geosci. Remote Sens. 60, 1–11. 10.1109/TGRS.2022.3165371

[B30] Zhao Y. Celik T. Liu N. Li H.-C. (2022). A comparative analysis of gan-based methods for sar-to-optical image translation. IEEE Geosci. Remote Sens. Lett. 19, 1–5. 10.1109/LGRS.2022.3177001

[B31] Zhu J.-Y. Park T. Isola P. Efros A. A. (2017). “Unpaired image-to-image translation using cycle-consistent adversarial networks,” in *Proceedings of the IEEE International Conference on Computer Vision* (Venice: IEEE), 2223–2232. 10.1109/ICCV.2017.244

